# Supplementary material for: Stability of local secondary structure determines selectivity of viral RNA chaperones
Source: Nucleic Acids Res. 2018 May 18;46(15):7924–37. doi: 10.1093/nar/gky394 (PMC6125681; doi:10.1093/nar/gky394)
Supplement: Supplementary Data [file gky394_supplemental_files.zip › SI_24.04.2018.pdf]

Supporting Data

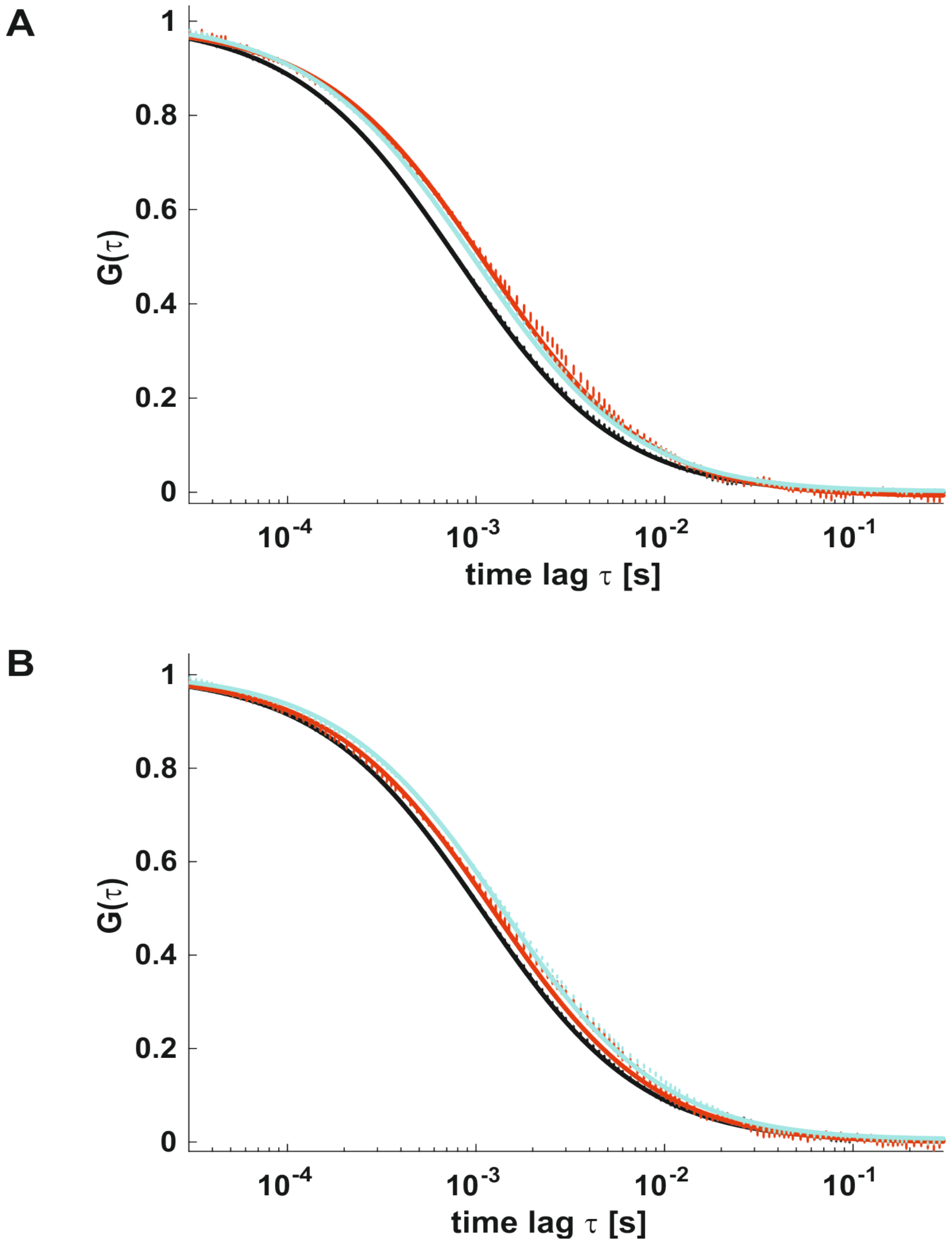

Supplementary Figure S1. NSP2 and σNS both bind ssRNAs S5 and S11

Normalized fluorescence autocorrelation functions (ACFs) corresponding to ATTO565-dye labelled ssRNA S5 (1610 nts, panel A) and ATTO647-dye labelled ssRNA S11 (667 nts, panel B) from the same data sets used for cross-correlation analysis in Figure 1, i.e. under identical conditions (equimolar mixtures of labelled RV RNAs S5 and S11, 55 nM each, were incubated alone (black), and with 5 μM of NSP2 (red) or σNS (cyan) and diluted to achieve 1 nM of each RNA). Given the large initial size of either RNA, the change in diffusion coefficient upon protein binding is minor, and more prominent for a smaller ssRNA S11 (667 nts). The observed slower diffusion of S11 RNA in complex with NSP2 (B, shown in red) likely reflects S11:S5 complex formation, which results in increase of its size.

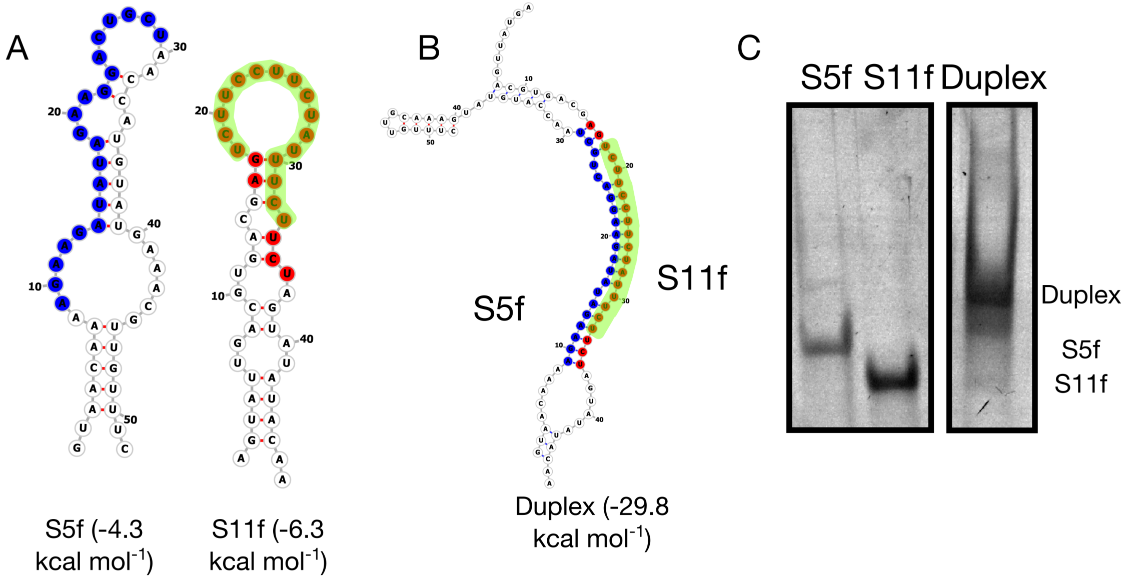

Supplementary Figure S2. Spontaneous annealing of the RNA fragments derived from S5 and S11 RNAs (S5f and S11f, respectively)

A: Rotavirus genomic ssRNA fragments spanning S5 (S5f, 299 – 350) and S11 (S11f, 31 – 77). Regions involved in sequence-specific RNA-RNA interaction are coloured in blue and red. Sequence of S11f corresponding to 17mer RNA used in the RNA-RNA interaction assay shown in Figure 1B is highlighted in green.

B: Inter-molecular duplex formed between S5f and S11f ssRNAs, schematically shown in (A).

C: Native 15% PAGE of S5f and S11f ssRNAs. Staining – SYBR gold. Left – S5f and S11f ssRNA fragments alone. Right – Spontaneously annealed S5f and S11f after co-incubation for 15 minutes at 37°C (10 nM each strand, 100 mM NaCl, 10 mM HEPES-Na, 1 mM $MgCl_2$ pH 7). RNAs were visualised by staining with SYBR gold and imaged by scanning with 488 nm excitation.

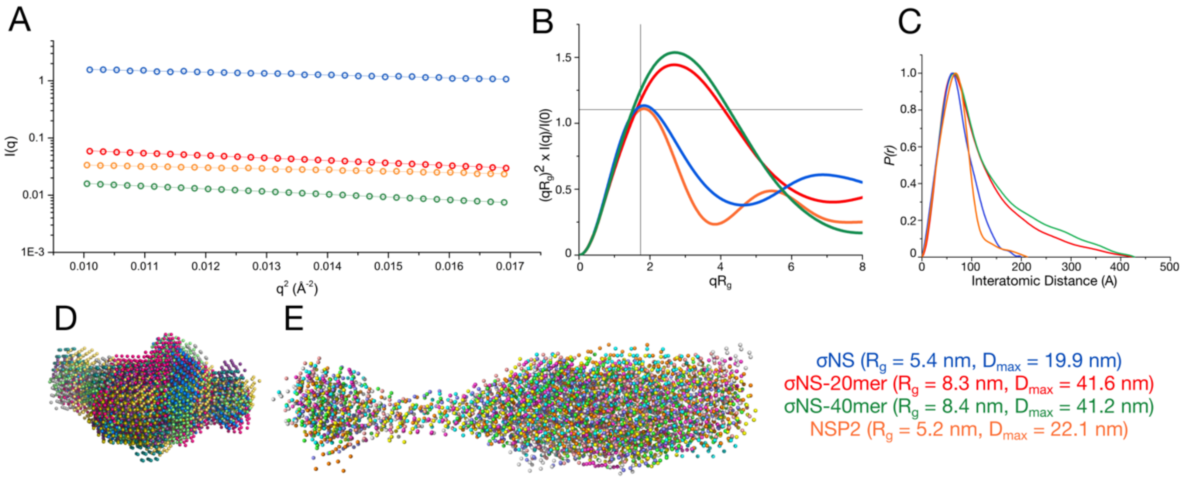

Supplementary Figure S3. SAXS characterisation of σNS-RNP complex

A: Guinier region analysis of σNS and NSP2 apoproteins (blue and orange, respectively) and σNS-RNPs (20-mer RNA: red, 40-mer RNA: green). All samples are monodisperse.

B: Dimensionless Kratky plot of σNS apoprotein (blue) and σNS-RNP formed with a 20 nt ssRNA (red), σNS-RNP formed with a 40 nt ssRNA (green). Octameric NSP2 apoprotein is shown for comparison (orange). The cross-hairs denote the Guinier-Kratky point (1.732, 1.104), the peak position for an ideal, globular particle (1). Both σNS apoprotein and NSP2 are globular, whereas σNS-RNP complexes with either 20mer or 40mer RNAs are less compact, more extended and more flexible, as is indicated by the upwards-right shift (2).

C: Pairwise Distance Distribution Function plots of σNS apoprotein (blue) and RNP complexes assembled using 20-mer (red) and 40-mer (green) ssRNA. Upon RNA binding, both the D_{max} and R_g values do not significantly increase (<1%), suggesting that under these conditions σNS adopts the same oligomeric state in the presence of either RNA, and does not further increase from an octamer as the length of RNA increases. NSP2 (orange) has a R_g value of 5.2 nm, consistent with the size of NSP2 octamer.

D & E: Best representative dummy atom models of σNS apoprotein (D) and RNP-20-mer complex (E) that were used to generate averaged and filtered SAXS models (Figures 4 A & B). The ensemble resolution of either model as estimated by SASRES were 65 ± 5 Å (D) and 51 ± 4 Å (E) and had normalized spatial discrepancies (NSD ± SD) of 0.820 ± 0.05 (D) and 0.576 ± 0.03 (E), as estimated by SUBCOMP (3). Both models describe the scattering data well (final χ^2 against raw data were 1.6 for *D* and 1.2 for *E*).

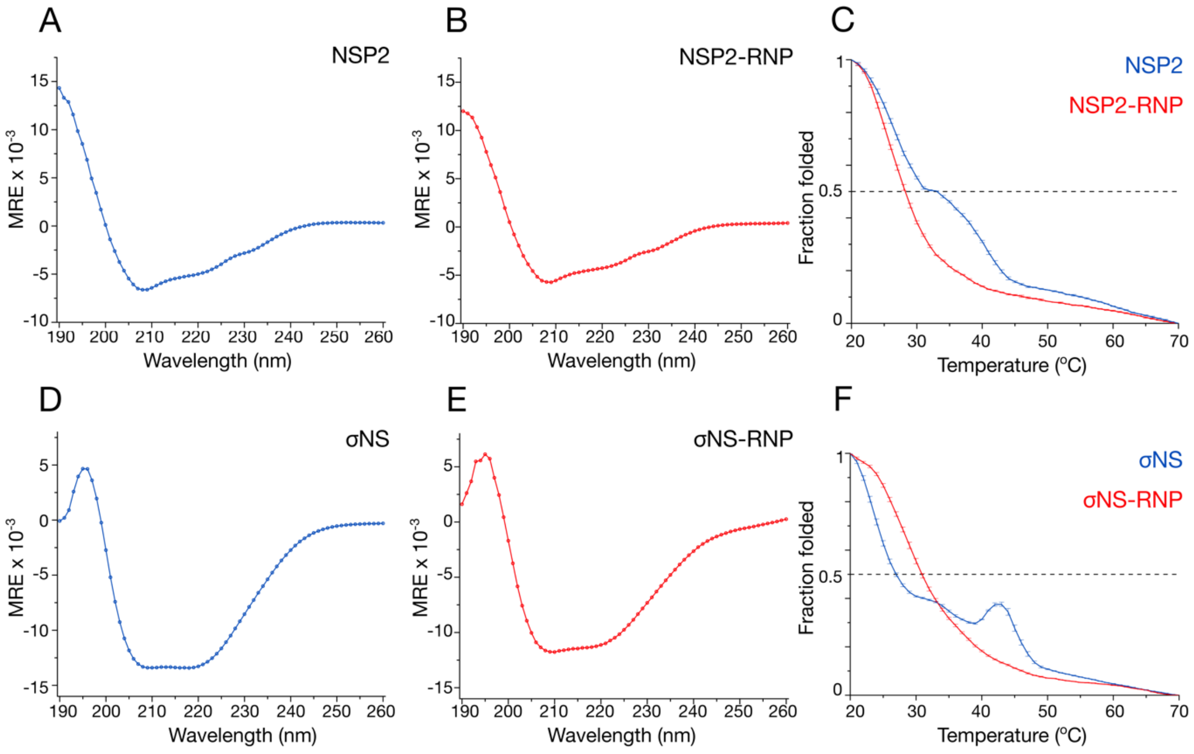

Supplementary Figure S4. Stabilities of NSP2 and σNS, and their RNP complexes.

Circular Dichroism (CD) spectra of NSP2 (upper panel) and σNS (lower panel) in the absence (blue), and presence of RNA (red). Spectra are shown in units of Mean Residue Ellipticity (MRE). Protein and RNP samples were subject to thermal denaturation, as described in Materials and Methods. Thermal melt curves of NSP2 (C) and σNS (F) in the absence (blue) and presence (red) of RNA. Fraction folded corresponds to the change in Θ_{222nm} as a function of temperature. Measurements were taken at 1°C temperature increments between 20°C and 70°C. Fluctuations in fraction folded for NSP2 and σNS apoprotein are likely due to aggregation during thermal denaturation.

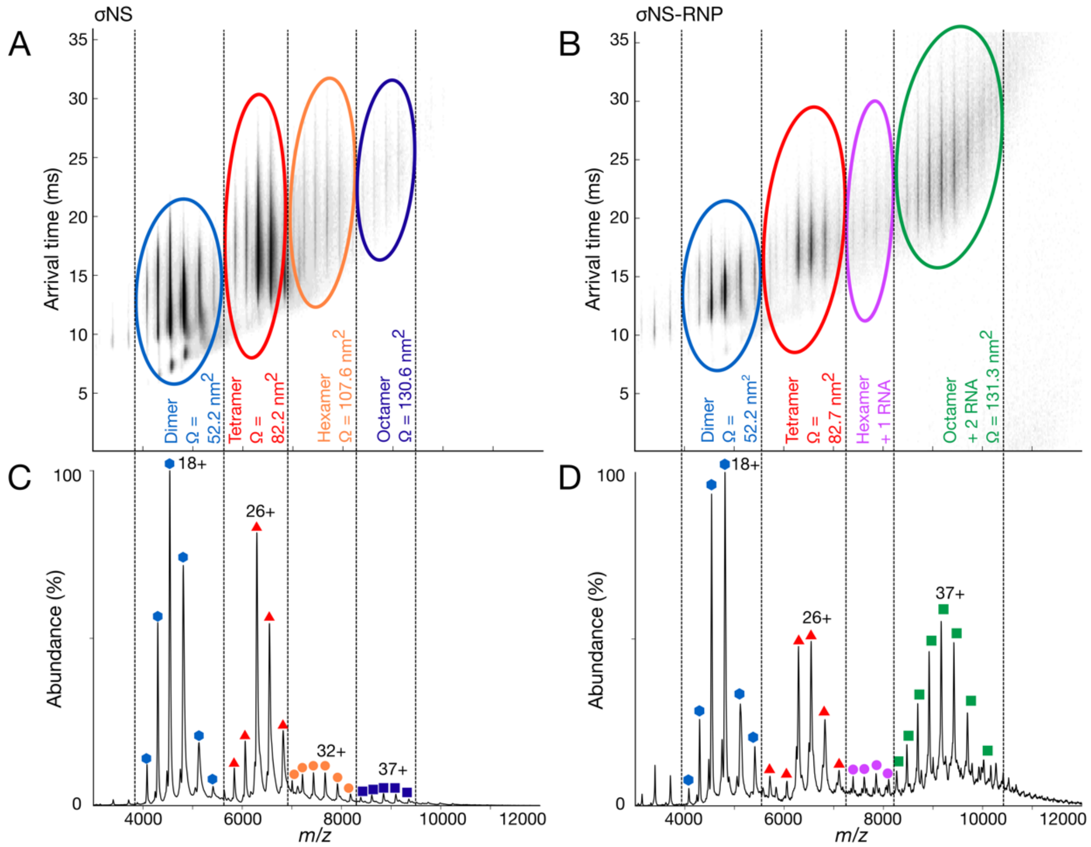

Supplementary Figure S5. ESI mass-spectra of σNS and σNS-RNP.

A & B: ESI-IMS-MS drift plots of σNS apoprotein (A) and RNP complex (B). Averaged collisional cross-sectional (CCS) areas (Ω) of each detected oligomeric species are shown in nm^2.

C & D: Cumulative m/z spectra of σNS apoprotein (C) and RNP complex (D). The most abundant charge state is labelled for each species detected. Different oligomers are indicated by different coloured ovals (dimer – blue, tetramer – red, hexamer – orange, octameric apoprotein – dark blue, hexamer + 1 RNA – magenta, octamer + 2 RNA – green). Observed masses of each oligomer and charge state are summarised in **Supporting Tables S2 & S3**. We were unable to derive CCS values for a hexameric RNP complex due to its consistently low abundance in the ESI spectrum.

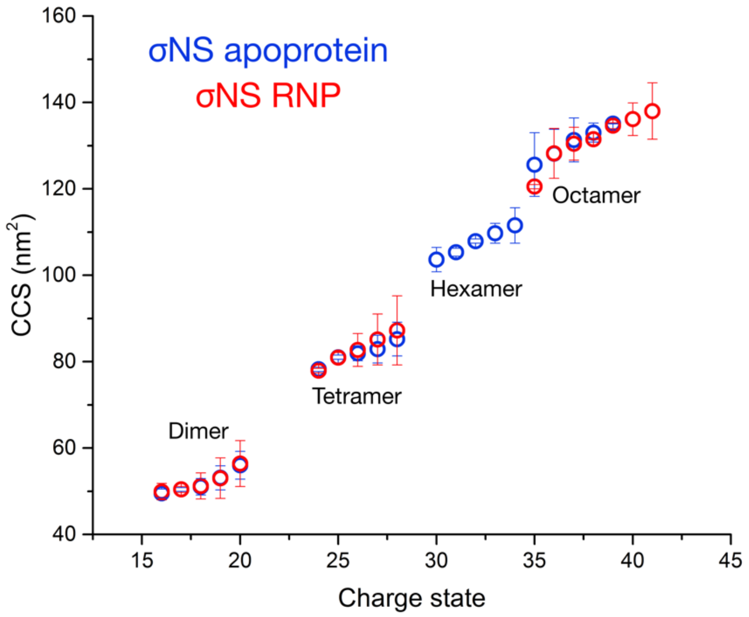

Supplementary Figure S6. Collisional cross-sections (CCS) of oligomers present in σNS and RNP samples, examined by ESI-IMS-MS.

Plot of observed CCSs of oligomers as a function of charge state. CCS values are reported as mean±SD from two independent experiments. The extremely low abundance of hexameric RNP species precluded their sizing in IMS mode.

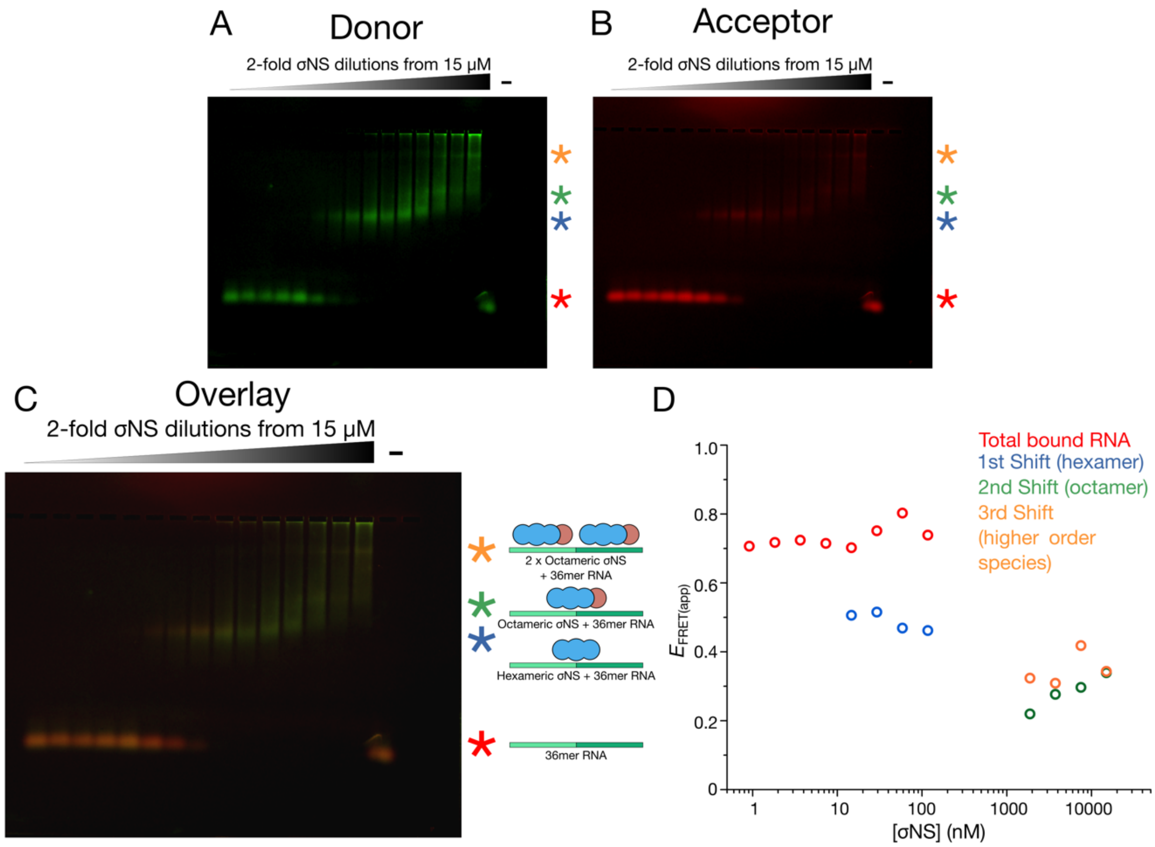

Supplementary Figure S7. σNS binding and unwinding of the RNA stem-loop, examined by in-gel FRET

A: Native 1.5% agarose gel (as shown in Figure 5B) excited with 532 nm laser, donor only imaged (shown in green).

B: Native agarose gel as described above, with acceptor only imaged (shown in red).

C: Overlay of donor only and acceptor only gel images, shown in the upper panel. Free RNA (red asterisk) has high acceptor emission, while σNS-bound RNA (blue, green and orange asterisks) has higher donor emissions, indicating RNA stem-loop disruption upon protein binding. RNP complexes corresponding to the band-shifts observed on a gel are shown schematically on the right.

D: Apparent FRET efficiencies of each RNP species separated on a native gel. Higher σNS concentration yields a larger fraction of the unfolded, σNS-bound stem-loop.

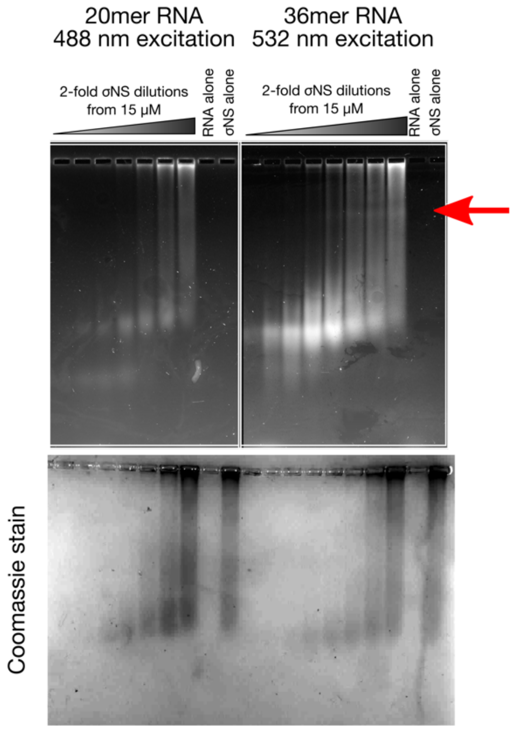

Supplementary Figure S8. Multiple σNS oligomers can bind 36-mer RNA stem-loop.

Metastable 20-mer AlexaFluor 488-labelled RNA ("20mer," left) and a dual-labelled 36-mer hairpin ("36mer," right) were incubated with increasing amounts of σNS. At high σNS concentrations (>2 μM), an additional shift appears (red arrow) when incubated with 36mer RNA, but not 20mer RNA. As σNS binds RNA with a ~20 nt footprint (4, 5), this shift is consistent with two σNS oligomers bound to the same 36mer RNA. Furthermore, such shift is not observed with the 20mer RNA. Coomassie staining confirms presence of proteins in the band. Note that free RNA was deliberately run off the gel to ensure sufficient separation of higher order species (for further details see **Figure 5** and **Supplementary Figure S7**). Due to the high isoelectric point of NSP2 (pI = 9), such a gel shift assay could not be carried out with NSP2 under similar conditions.

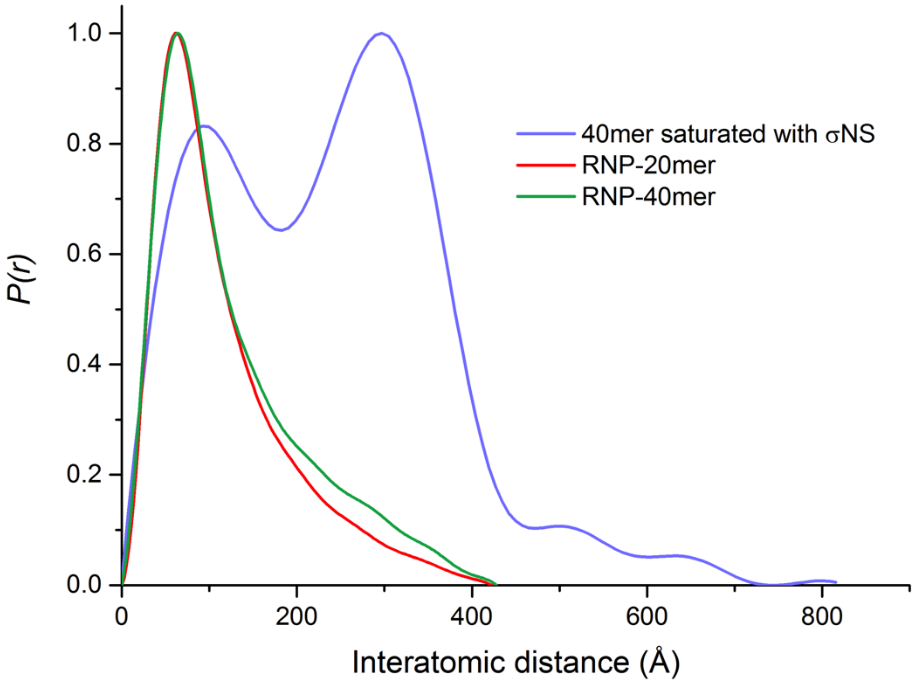

Supplementary Figure S9. Saturation of 40-mer ssRNAs with σNS results in larger, polydisperse species

Pairwise distance-distribution functions (PDDFs) of σNS in complex with 20mer and 40mer RNA (red and green, respectively), where RNP complexes are formed using stoichiometric excess of RNA over σNS oligomers, allowing efficient octamer formation. Saturation of 40mer RNA with σNS results in two distinct PDDF maxima (blue), suggesting concurrent binding of two σNS oligomers to the same RNA, consistent with the observed band shifts with a 36mer stem-loop (Supplementary Figure S8).

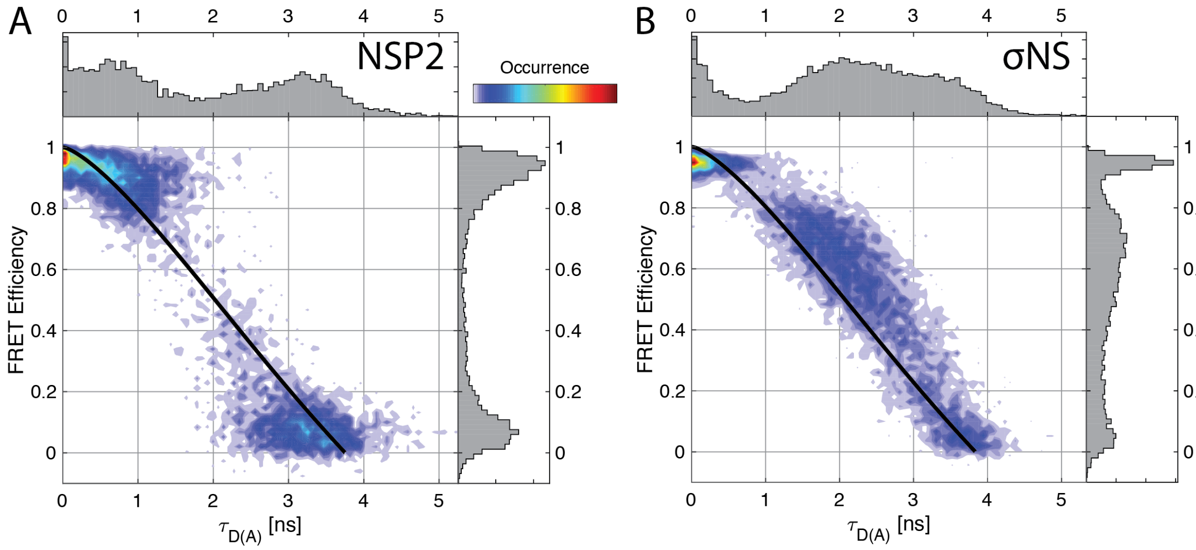

Supplementary Figure S10: Conformational dynamics of the RNA stem-loop monitored in the spFRET experiments.

Two-dimensional histograms of the FRET efficiency versus the fluorescence lifetime of the donor fluorophore, $\tau_{D(A)}$, for RNA stem-loop bound to NSP2 (A) and σNS (B). FRET efficiency and donor fluorescence lifetime are related by $E = 1 - \frac{\tau_{D(A)}}{\tau_{D(0)}}$, where $\tau_{D(A)}$ and $\tau_{D(0)}$ are the fluorescence lifetimes of the donor in the presence and absence of the acceptor fluorophore and $\tau_{D(0)}$ can be determined from the subpopulations of molecules lacking the acceptor fluorophore. Populations of static molecules, i.e., not showing conformational dynamics during the transit through the confocal volume of ~1 ms duration, are described by the polynomial static FRET line, shown as solid black line. RNA molecules undergoing conformational dynamics on the timescale of μs-ms deviate from this line (6). For NSP2, no significant deviation from the static FRET line is observed. In contrast, the intermediate FRET population bound to σNS deviates from the static FRET line, revealing sub-millisecond conformational dynamics of σNS-bound RNA stem-loops.

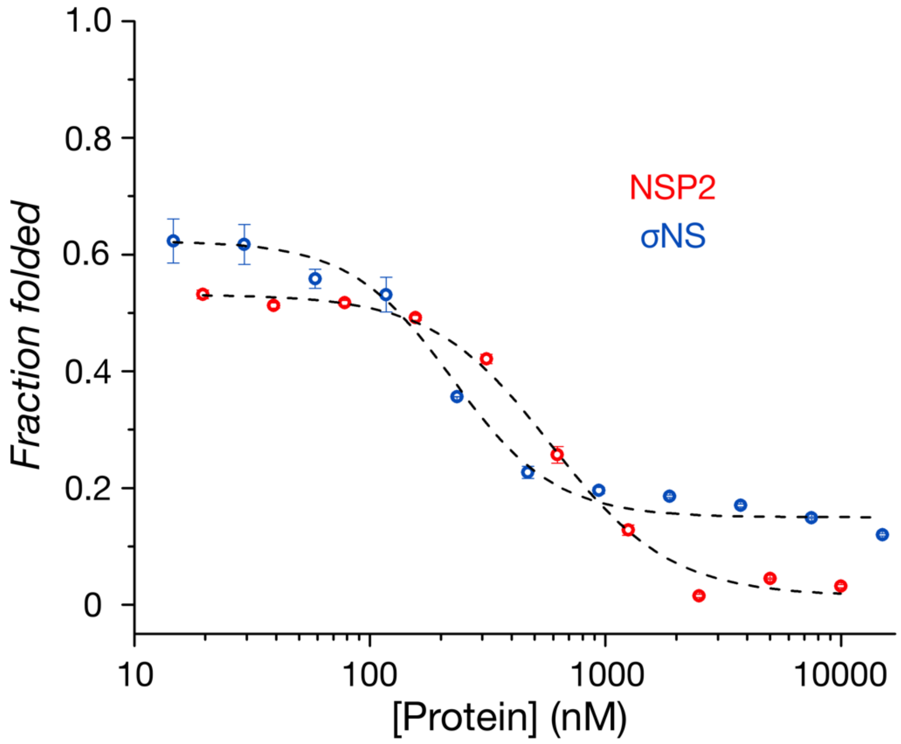

Supplementary Figure S11. RNA helix destabilization by NSP2 and σNS, monitored by ensemble FRET

Apparent FRET efficiencies for fully folded (‘Fraction folded’ = 1) and formamide-denatured RNA stem-loops (‘Fraction folded’ = 0) were measured and compared to the apparent FRET efficiencies observed in the presence of σNS (blue) or NSP2 (red). Titrations of NSP2 and σNS into 36mer RNA stem-loop (10 nM, see Materials and Methods) result in decrease of the apparent FRET efficiency, consistent with protein-assisted helix unwinding.

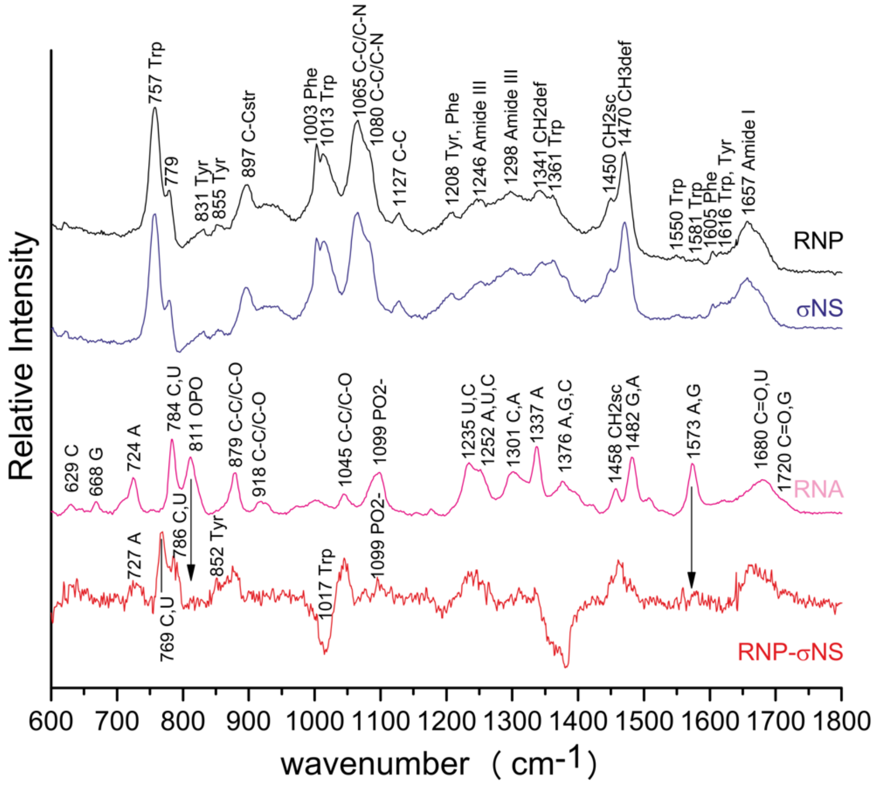

Supplementary Figure S12. σNS binding destabilizes A-form RNA

Raman difference spectrum of σNS-bound stable 20-mer RNA hairpin. σNS alone spectrum (blue) was subtracted from that of the ribonucleoprotein (RNP, black) to obtain a difference spectrum corresponding to the bound RNA (red). Spectrum of the protein-free RNA hairpin is shown in magenta. Selected bands are labelled with wavenumbers and assignments (designated by one letter base abbreviation and three letters for amino acids. Vertical arrows indicate prominent changes in the spectrum of the bound RNA compared to the free RNA. Note that since RNA contribution to RNP is weak, all RNA spectra are scaled 20 fold to aid comparison. A-form exhibits a prominent band at 810 cm^{-1}, which is assigned to symmetric stretching vibration of the phosphodiester linkage (7, 8). This feature is absent in the spectra of unfolded form or shifts to ~830 cm^{-1} in the B-form.

Supporting Tables

RNA substrate	Sequence (3' – 5')	ΔG of folding (kcal mol^{-1})*
Unstructured (unlabelled)	CUUUUCAAGACAUGCAACAA	0
Unstructured	Alexa488-CUUUUCAAGACAUGCAACAA	0
Metastable	Alexa488-CUUCUUUCGAAAUUAAGAAG	-3.8
Stable	Alexa488-UCUUUGCAAACAUGCAAAGA	-8.1
40mer	CUUUUCAAGACAUGCAACAA<br>CUUUUCAAGACAUGCAACAA	-1.6
Dual-labelled stem-loop	ATTO532-AAAUCUUUGCAAACU<br>AUCCAAUCCAUGCAAAGAUAA-ATTO647N	-8.9
Cy3 17mer	Cy3-UUCCUUCUAUUUCUUCU	0
Cy5 17mer	Cy5-UUCCUUCUAUUUCUUCU	0
RV Segment 5	S5: KF729657.1	0
RV Segment 11	S11: KF729697.1	0
S5f	GUAACAAA**AGAAGAUAUAGAAGG**<br>**AC**UGCUAACCAUGUAUGAAACGUUGUUUC	-4.5
11f	AGUAUUGACGUGACGAGUC**UUCC**<br>**UUCUAUUUCUUCU**AGUAUAUACAA	-6.3

Supporting Table S1 Sequences of the RNAs used in this study. *Minimum folding energies (MFEs) were calculated using Vienna RNA server (9).

	RNA substrate	$K_{d(obs)}$ (nM)	ΔG_{obs} (kcal mol^{-1})	$K_{d(obs)}$ (nM) + 10 mM $MgCl_2$	ΔG_{obs} (kcal mol^{-1}) + 10 mM $MgCl_2$	$\Delta\Delta G_{obs}$ (kcal mol^{-1})
NSP2	Unstructured	20.7±1.67	-10.12 ± 0.65	28 ± 1.48	-9.94 ± 0.67	0.19
	Metastable	20.6±3.20	-10.12 ± 0.63	212 ± 21.81	-8.79 ± 0.66	1.32
	Stable	20.8±1.8	-10.12 ± 0.65	629 ± 47.12	-8.16 ± 0.68	1.97
σNS	Unstructured	37±1.48	-9.91 ± 0.67	45.3 ± 0.69	-9.67 ± 0.71	0.23
	Metastable	24± 2.58	-10.03 ± 0.64	420 ± 14.33	-8.39 ± 0.70	1.64
	Stable	137± 1.94	-9.03 ± 0.73	1283 ± 95.76	-7.76 ± 0.68	1.28

Supporting Table S2 Overall free energies of NSP2 and σNS binding to 20mer RNAs, calculated from binding curves in Figure 7. $\Delta\Delta G_{obs}$ corresponds to the difference in free energies of binding in the presence and absence of 10 mM $MgCl_2$. Further details – see Materials and Methods.

	NaCl (mM)	$K_{d(obs)}$ (nM)	ΔG_{obs} (kcal mol^{-1})	ΔG_{PE} (kcal mol^{-1})	ΔG_{nPE} (kcal mol^{-1})	% Electrostatic contribution
NSP2	50	12 ± 0.49	-10.43 ± 0.67	-2.62 ± 0.63	-7.81 ± 0.63	25.1
	100	21 ± 0.95	-10.05 ± 0.67	-2.01 ± 0.63	-8.04 ± 0.63	20.0
	150	63 ± 6.18	-9.50 ± 0.68	-1.66 ± 0.63	-7.84 ± 0.63	17.5
	200	98 ± 8.69	-9.23 ± 0.66	-1.41 ± 0.63	-7.82 ± 0.63	15.3
	1000*	N/A	-7.89*	0	-7.89*	0*
σNS	50	29.7 ± 1.10	-9.91 ± 0.68	-4.57 ± 0.64	-5.34 ± 0.64	46.1
	150	58 ± 4.63	-9.53 ± 0.66	-2.90 ± 0.64	-6.63 ± 0.64	30.4
	250	168 ± 29.09	-8.92 ± 0.49	-2.12 ± 0.64	-6.80 ± 0.64	23.7
	350	421 ± 22.25	-8.39 ± 0.64	-1.60 ± 0.64	-6.79 ± 0.64	19.1
	500	1470 ± 100.9	-7.68 ± 0.69	-1.06 ± 0.64	-6.62 ± 0.64	13.8
	1000*	N/A	-6.71*	0	-6.71*	0*

Supporting Table S3 Electrostatic contributions to free energy of binding of NSP2 and σNS, as calculated from binding curves measured in Figure 8. * denotes values estimated through extrapolation of binding energies to 1 M NaCl, where electrostatic component of free energy of binding is eliminated (10), further details – see Materials and Methods.

	m/z	Charge state	CCS (nm^2)	Predicted mass (Da)	Calculated mass (Da)
Dimer	5124	16	49.5 ± 1.0	81965	81903
	4822	17	50.4 ± 0.4		
	4555	18	51.0 ± 1.9		
	4315	19	53.1 ± 2.8		
	4099	20	56.0 ± 3.2		
Tetramer	6831	24	78.2 ± 0.5	163930	164007
	6558	25	81.0 ± 0.5		
	6306	26	81.9 ± 1.7		
	6072	27	82.9 ± 3.2		
	5856	28	85.2 ± 3.9		
Hexamer	8197	30	103.6 ± 2.8	245895	245924
	7933	31	105.3 ± 0.9		
	7685	32	107.9 ± 0.5		
	7452	33	109.7 ± 2.3		
	7233	34	111.53 ± 4.1		
Octamer	9368	35	125.6 ± 7.4	327860	328011
	9108	36	128.1 ± 5.7		
	8862	37	131.3 ± 5.1		
	8629	38	133.0 ± 2.2		
	8408	39	135.1 ± 0.1		

Supporting Table S4 σNS apoprotein oligomers m/z, charge states, collision cross-sections and masses estimated by ESI-IMS-MS. CCSs are reported as mean ± SD of two independent experiments.

	m/z	Charge state	CCS (nm^2)	Predicted mass (Da)	Calculated mass (Da)
Dimer	5124	16	49.9 ± 1.9	81965	81903
	4822	17	50.4 ± 0.7		
	4555	18	51.2 ± 3.0		
	4315	19	53.0 ± 4.7		
	4315	20	56.4 ± 5.3		
Tetramer	6831	24	77.9 ± 0.5	163930	164007
	6558	25	80.9 ± 1.2		
	6306	26	82.7 ± 3.8		
	6072	27	85.1 ± 5.9		
	5856	28	87.2 ± 8.0		
Octamer + 2 RNA	9710	35	120.5 ± 0.5	341460	339804
	9441	36	128.2 ± 5.8		
	9185	37	130.4 ± 3.8		
	8944	38	131.5 ± 0.1		
	8714	39	134.6 ± 0.7		
	8497	40	136.1 ± 3.8		
	8289	41	138.0 ± 6.5		

Supporting Table S5 - σNS RNP oligomers m/z, charge states, collision cross-sections and masses estimated by ESI-IMS-MS. CCSs are reported as mean ± SD of two independent experiments. Note that CCS values for hexameric RNP complex could not be estimated due to low intensities.

References

1. Rambo,R.P. and Tainer,J.A. (2011) Characterizing flexible and intrinsicallyunstructured biological macromolecules by SAS using the Porod-Debye law. *Biopolymers*, **95**, 559–571.
2. Peng, Yi, Curtis,J.E., Fang,X. and Woodson,S.A. (2014) Structural model of an mRNA in complex with the bacterial chaperone Hfq. *Proc Natl Acad Sci U S A*, **111**, 17134–9.
3. Kozin,M.B. and Svergun,D.I. (2001) Automated matching of high- and low-resolution structural models research papers Automated matching of high- and low-resolution structural models. *J. Appl. Crystallogr.*, **34**, 33–41.
4. Borodavka,A., Ault,J., Stockley,P.G. and Tuma,R. (2015) Evidence that avian reovirus σNS is an RNA chaperone: implications for genome segment assortment. *Nucleic Acids Res.*, **43**, 7044–7057.
5. Gillian,A.L., Schmaechel,S.C., Livny,J., Schiff,L.A. and Nibert,M.L. (2000) Reovirus protein sigma NS binds in multiple copies to single stranded RNA and shares properties with single stranded DNA binding proteins. *J. Virol.*, **74**, 5939–5948.
6. Kalinin, S., A. Valeri, M. Antonik, S. Felekyan, and C.A.M. Seidel. 2010. Detection of structural dynamics by FRET: a photon distribution and fluorescence lifetime analysis of systems with multiple states. *J. Phys. Chem. B.* **114**, 7983–7995.
7. Erfurth,S.C., Kiser,E.J. and Peticolas,W.L. (1972) Determination of the backbone structure of nucleic acids and nucleic acid oligomers by laser Raman scattering. *Proc. Natl. Acad. Sci. U. S. A.*, **69**, 938–941.
8. Lafleur,L., Rice,J. and Thomas,G.J. (1972) Raman Studies of Nucleic Acids. *Biopolymers*, **11**, 2423–2437.
9. Gruber,A.R., Lorenz,R., Bernhart,S.H., Neuböck,R. and Hofacker,I.L. (2008) The Vienna RNA websuite. *Nucleic Acids Res.*, **36**, 70–74.
10. Samatanga,B., Cléry,A., Barraud,P., Allain,F.H.-T. and Jelesarov,I. (2017) Comparative analyses of the thermodynamic RNA binding signatures of different types of RNA recognition motifs. *Nucleic Acids Res.*, **45**, 6037–6050.
